# Validation of Two Portion Size Estimation Methods for Use with the Global Diet Quality Score App

**DOI:** 10.3390/nu17091497

**Published:** 2025-04-29

**Authors:** Mourad Moursi, Marieke Vossenaar, Joanne E. Arsenault, Winnie Bell, Mario Chen, Megan Deitchler

**Affiliations:** 1 Intake—Center for Dietary Assessment, FHI 360, Washington, DC 20037, USAjarsenault@fhi360.org (J.E.A.); wbell@fhi360.org (W.B.); mdeitchler@fhi360.org (M.D.); 2 Biostatistics and Data Sciences, FHI 360, Durham, NC 27701, USA; mchen@fhi360.org

**Keywords:** GDQS app, portion size estimation, cubes, playdough, validation, weighed food records

## Abstract

**Background/Objectives**: The Global Diet Quality Score (GDQS) was developed to provide crucial information on diet quality. The GDQS app standardizes the collection of GDQS data using portion size estimation at the food group level with 3D cubes of pre-defined size. Playdough was proposed as a possible alternative method. This validation study assessed whether the GDQS obtained using cubes or playdough with the GDQS app was equivalent to the GDQS estimated by weighed food records (WFRs) for the same 24 h reference period. **Methods**: We used a repeated measures design where 170 participants aged 18 years or older estimated portion sizes using both the WFR and the GDQS app with cubes and playdough. To assess the equivalence between the GDQS-WFR and GDQS-cubes or GDQS-playdough, we utilized the paired two one-sided t-test (TOST), with 2.5 points pre-specified as the equivalence margin. We used the Kappa coefficient to quantify agreement between WFR, risk of poor diet quality outcomes, and food group consumption using the cubes or playdough. **Results**: GDQS-WFR, GDQS-cubes, and GDQS-playdough were all equivalent within the pre-specified 2.5-point margin (*p* = 0.006 for cubes and *p* < 0.001 for playdough). The cubes (κ = 0.5685, *p* < 0.0001) and playdough (κ = 0.5843, *p* < 0.0001) showed moderate agreement with WFR when classifying individuals at risk of poor diet quality outcomes. There was substantial to almost perfect agreement between the cubes and playdough methods and WFR for 22 out of the 25 GDQS food groups. Liquid oils exhibited the lowest agreement (κ = 0.059, 27.7% agreement, *p* = 0.009). **Conclusions**: Using the GDQS app with cubes or playdough was equivalent to the WFR in assessing diet quality as measured by GDQS. These findings contribute to the growing body of research, validating simplified tools for dietary assessment and paving the way for more frequent evaluations of diet quality.

## 1. Introduction

Nutritious diets are crucial for overall health, play a vital role in malnutrition globally [1], and provide lasting benefits to education outcomes and lifetime earnings [2]. Dietary patterns influence various aspects of health, including child growth, cardiovascular diseases, diabetes, cancer, and obesity. Recent global analyses consistently rank suboptimal dietary practices as the highest contributors to morbidity and mortality worldwide [1]. Unhealthy diets intersect with socioeconomic, environmental, and cultural determinants, emphasizing their central role in public health interventions aimed at reducing disease burdens and promoting long-term population health [3]. Measuring diet quality is crucial for informing policies and programs that promote healthy eating. To provide actionable, evidence-based information, dietary data must identify specific aspects of the diet requiring improvement within a population.

The Global Diet Quality Score (GDQS) is a metric of diet quality for global use developed and validated by the Intake—Center for Dietary Assessment at FHI 360, the T.H. Chan School of Public Health at Harvard University, and the National Institute of Public Health (INSP), Mexico [4]. The GDQS has several innovative features. It is designed to be sensitive to both nutrient adequacy and diet-related non-communicable disease (NCD) risk outcomes in diverse settings. It comprises two sub-metrics: the GDQS positive includes food groups such as eggs and legumes that are key sources of nutrients [5,6,7,8], while the GDQS negative comprises food groups such as processed meats and sugar-sweetened beverages that are known to have negative health effects [9,10,11,12]. The metric is entirely food-based and does not require the use of a food composition table for analysis. Unlike other diet quality metrics, the GDQS uses quantity of consumption information at the food group level expressed as low, medium, high, and very high to score the consumption of 25 food groups [13]. Population-based cut-offs have been identified to allow for reporting the percent of the population at high (GDQS < 15), moderate (GDQS ≥ 15 and <23), and low risk (GDQS ≥ 23) for poor diet quality outcomes [14].

In 2020, Intake developed a mobile application to simplify and standardize the collection and tabulation of the GDQS (the GDQS app). The GDQS app was designed to overcome many of the known operational challenges associated with collecting high-quality population-based data on simple diet-related metrics across different countries and contexts [15]. The use of the quantity of consumption information at the food group level in the scoring of the GDQS is an especially notable innovation in the construction of the metric in comparison to other simple diet quality-related metrics [13]. The GDQS app is used in conjunction with a physical set of ten 3D printed cubes of pre-defined sizes to operationalize portion size estimation. The volume of each cube was determined using the gram cut-offs associated with each food group in the GDQS metric, along with data on the density of the foods, beverages, and ingredients belonging to each food group [15]. The cubes are currently the standard method for portion size estimation when using the GDQS app.

Recognizing that estimating portion size in dietary surveys is challenging and that it may not be possible to obtain 3D-printed cubes in some settings, we propose a second method for portion size estimation as a possible alternative for use with the GDQS app. Playdough has been used in several dietary assessment studies and national surveys because it provides a flexible, interactive, and easy-to-use method for estimating a wide range of foods, including oddly shaped and amorphous foods [15]. Playdough has traditionally been used to estimate the amounts of individual foods and mixed dishes consumed [16,17], rather than the total amount consumed at the food group level, as would be the case if used in conjunction with the GDQS app. We are not aware of any studies that have tested participants’ ability to quantify consumption amounts with playdough at the food group level. However, several studies have shown playdough to be a valid assessment method at the individual food level [18,19]. In these studies, the assessment of the comparison between images, playdough, and household utensils showed no difference in accuracy among them.

The primary aim of this study was to assess whether the GDQS metric obtained by either using cubes or playdough as the portion size estimation method with the GDQS app provided equivalent data to the GDQS metric estimated by weighed food records (WFRs) for the same 24 h reference period. 

## 2. Materials and Methods

### 2.1. Study Design

This validation study was carried out in Washington, DC, USA, from November 2022 to June 2023 with 170 male and female participants. We used a convenience sample approach for the study, as statistically representative data were not needed to achieve the study objectives. Some aspects of the study were specifically designed to be appropriate for the COVID-19 environment, as planning for the study began in 2021, at the height of the pandemic. We planned to enroll 200 participants. The intended sample size was based on observed sample sizes from similar studies [18,20,21] and logistical limitations of the study. A post-hoc power analysis indicated that a sample size of 170 provided sufficient statistical power (>80%) for testing the primary equivalence of the GDQS metric estimated by the WFR and the GDQS app with the two portion size estimation methods (cubes and playdough).

The validation study used a repeated measures design in which each participant estimated portion sizes using the following: (1) WFR; (2) GDQS app interview with estimation by two methods, cubes and playdough. The GDQS app was designed to randomize the order in which cubes or playdough were used as a portion size estimation method. The full interview with the GDQS app was completed using one portion size estimation method before the respondent was asked to estimate portion sizes using the other method.

Each participant was engaged in the study over three consecutive days. On the first day, participants came to the FHI 360 office and were given an in-person training session on how to weigh foods, beverages, and mixed dishes. We gave each participant a calibrated digital dietary scale and WFR data collection forms. On the second day, participants weighed and recorded all foods, beverages, and mixed dishes during a 24 h period. On the third day, participants returned to the FHI 360 office to submit the completed WFR forms, complete a face-to-face GDQS app interview with both portion size estimation methods, and provide feedback on using the cubes and playdough.

### 2.2. Recruitment of Participants

We recruited participants by email and by posting flyers. We sent emails to the neighborhood and university listservs. We posted flyers at local cafes, coffee shops, supermarkets, and universities in Northwest DC. We included all participants who fit the eligibility requirements in the study.

As an eligibility requirement, participants had to be 18 years or older, fully vaccinated against COVID-19 (self-reported), confirm that they did not plan to fast during the 24 h reference period, and be fluent in English or Spanish. Since the study primarily focused on comparing portion estimation methods and did not intend to describe diets in this specific population, intermittent fasting was allowed. In addition, participants had to agree not to eat mixed dishes (i.e., dishes composed of several ingredients) prepared outside of home, or for which they did not know or could not weigh the ingredients during the 24 h reference period for which they were asked to record and weigh the foods they consumed.

### 2.3. Ethical Considerations

FHI 360’s Office of International Research Ethics reviewed and approved the study. All study participants provided written informed consent. At the conclusion of all data collection activities, we provided the participants a $200 electronic gift card to compensate for their time required over the 3 days of data collection activities and any travel and/or parking costs incurred to participate in the two in-person data collection activities at the FHI 360 office.

### 2.4. Estimating Amounts Using WFR

Participants received 40–60 min of in-person training in groups of up to five people on how to use a dietary scale and how to weigh all foods, beverages, and mixed dishes consumed, as well as the ingredients used in mixed dishes. We provided participants with a calibrated digital dietary scale (KD-7000, capacity 7 kg, MyWeigh, Phoenix, AZ, USA) accurate to 1 g and multiple copies of two types of paper data collection forms (the food form and the recipe form). We gave participants a WFR guide and videos as additional reference tools that could be accessed during the 24 h reference period. We also provided participants with emails and phone numbers to contact the study team in case they had any questions.

On the day of WFR data collection, participants used the food form to record the amounts served and any leftovers of foods, beverages, and mixed dishes consumed during the 24 h reference period. Participants weighed foods in a container, first placing the container on the scale and using the tare function to adjust to zero, then placing the food in the container to obtain the weight of the food in grams. If the participant consumed mixed dishes, the amounts of all ingredients used to prepare the dish were recorded on the recipe form (one form per reported recipe). Participants also recorded the weight of the prepared mixed dish by weighing the empty container before preparation and the total weight of the container and mixed dish after preparation. Enumerators checked all paper forms for completeness, clarity, and legibility upon submission by each participant, before beginning data collection with the GDQS app.

### 2.5. Administering the GDQS App Interview

A trained team of seven enumerators administered the GDQS app interview to study participants. The app uses 7 steps to collect key socio-demographic data and dietary data to tabulate GDQS [15]. Briefly, the GDQS app uses an open recall to collect a full list of food, beverages, and mixed dishes consumed the previous day and night. The respondent is then asked to report the main ingredients of mixed dishes and provide details about food items when this is needed to classify them into the corresponding GDQS food groups. Next, respondents are asked to report the consumption of deep-fried foods and caloric sweeteners. In the final step, the GDQS app automatically classifies all foods, beverages, and ingredients consumed into the corresponding GDQS food group for portion size estimation.

For this study, we inserted a verification step in which we used the WFR data as a reference to verify that the foods recalled as consumed by the participant during the GDQS app interview matched the foods recorded on the WFR forms. If there was a discrepancy between the two, the data collection team queried the participant to ensure that the correct amendments and notations were made to either the food items in the WFR or the food items recorded on the GDQS app, or both. These amendments were made primarily to clarify when participants named their foods differently between the WFR and the open recall in the GDQS app, or when a food item was recorded in the WFR but was missed during the open recall portion of the GDQS app interview. This verification step was implemented to ensure that the comparison of GDQS was based on the same foods and not influenced by errors of omission or intrusion of foods in the GDQS app.

### 2.6. Estimating Amounts with Cubes

In the final step, the enumerator read back to the participant all the foods belonging to the same GDQS food group. The list of food groups is automatically displayed in the app. The enumerator then asked the participant to visualize the combined amount of foods, beverages, and ingredients consumed within the same food group and to point to the cube that came closest in volume to the combined amount. This procedure was repeated for all the GDQS food groups reported, except for liquid oils.

### 2.7. Estimating Amounts with Playdough

The playdough used in this study was the commercial Play-Doh brand. For estimating the amount consumed with playdough, the final step asked participants to report amounts of each GDQS food group using the same list provided by the GDQS app and to visualize the total amount consumed for each food group. The enumerator asked the participant to mold playdough to represent the same volume of the combined amount for each GDQS food group, except liquid oils. The enumerator then weighed the playdough molded by the participant and recorded the gram amount in the GDQS app.

### 2.8. The Built-In Algorithm to Infer the Amount of Liquid Oils Consumed

We did not ask participants to estimate the amount consumed for the liquid oils food group because it is unrealistic to expect an individual to quantify accurately the amount of liquid oils consumed in mixed dishes. The GDQS app uses an algorithm to infer a category of consumption in which to classify the respondent for the liquid oils food group. The algorithm used in the GDQS app classifies a respondent at the highest consumption level for liquid oils if (i) any deep-fried foods prepared at home with liquid oils were consumed, or (ii) two or more mixed dishes were consumed, or (iii) liquid oils were poured on food or used in food preparation. If only one mixed dish is reported as consumed (and no deep-fried foods prepared at home with liquid oils were consumed, and liquid oils were not poured on food or used in food preparation), the GDQS app classifies the respondent in the medium consumption level for liquid oils. The GDQS app classifies all other respondents in the lowest consumption level for liquid oils.

### 2.9. Data Entry and Processing

The study team entered the WFRs data in Microsoft Access using a custom data entry table. We conducted extensive checks of the WFR data against paper records to ensure the accuracy of the data, and corrections were made in Access as needed. Mixed dishes were disaggregated into their corresponding ingredients. We calculated the proportions of ingredients in mixed dishes by dividing the weight of the ingredients by the total weight of the mixed dish after preparation. To calculate the amounts of ingredients consumed, we multiplied these proportions by the amount of mixed dish consumed. We applied yield factors [22] to convert raw weights into cooked weights, where applicable, depending on the type of mixed dish. Foods, beverages, and ingredients from the WFR were classified into their corresponding GDQS food group, amounts in grams were summed by GDQS food group, and the GDQS was tabulated using the corresponding gram cut-offs and point allocation [14].

Data collected with the GDQS app using cubes was automatically processed. The GDQS app outputs files with foods, beverages, and ingredients classified into their corresponding food groups, and with amounts consumed at the food group level assigned based on the cube selected by the participant for each food group consumed.

For playdough, the weight of the playdough, as entered into the app during data collection, was output, and then calculations were performed in Stata. Playdough weight was converted into an estimate of the total amount of foods, beverages, and ingredients reported as consumed for each GDQS food group in grams using information on the density of playdough along with the same average food group density value as used with the cubes. The formula used was the following: (playdough weight in grams/density of playdough) multiplied by average food group density equals grams of food group consumed.

### 2.10. Data Analysis

The two one-sided paired t-test for equivalence (TOST) was used to assess equivalence between the GDQS derived from WFR data and GDQS collected with the app using cubes or playdough [23]. A priori, we defined the acceptable equivalence margin, which is the range within which the difference in GDQS by WFR data collection and app data collection is considered negligible, as 2.5 GDQS points. We defined this equivalence margin by applying an equivalence margin of 20%, which was previously used to compare different dietary assessment data collection methods [21,24,25] to the range of GDQS values expected to be observed (10.5 to 23.0), as found in global secondary data analyses conducted by Intake [26]. The range of 12.5 × 0.20 equivalence margin equals 2.5 GDQS points.

The Kappa coefficient of agreement was used to quantify the level of agreement between WFR and cubes or playdough for GDQS categorical data. Outcomes assessed included percent at low, moderate, and high risk of poor diet quality outcomes, and percent at low, medium, and high (and very high, in the case of high-fat dairy) levels of consumption for the 16 GDQS positive food groups and the 9 GDQS negative food groups.

The Kappa coefficient ranges from −1 to 1, where 1 indicates perfect agreement, 0 suggests agreement no better than chance, and negative values imply disagreement greater than expected by chance. We used the interpretation of Kappa coefficients proposed by Landis and Koch [27], with values above 0.81 considered almost perfect agreement, 0.61–0.80 substantial, 0.41–0.60 moderate, 0.21–0.40 fair, and 0.00–0.20 none to slight. We also present results for the test of whether the estimated Kappa coefficient was different from zero. Statistical significance was assessed at the two-sided 5% level. *p*-values < 0.05 indicate that the agreement is not due to chance.

All statistical analyses were conducted using Stata 16.1 (Stata Corporation, College Station, TX, USA) with the TOST package version 3.1.4 installed [28]. We verified TOST equivalence tests using SAS 9.4. (SAS Institute Inc., Cary, NC, USA). Results obtained by Stata 16.1 and SAS 9.4 were identical.

## 3. Results

### 3.1. Sample Characteristics

Demographic characteristics of the study population are provided in Table 1. More than two-thirds of participants (69%) were aged between 18 and 35 years. The sample was predominantly female (71%). Approximately half of the study participants identified as white (51%), one quarter (24%) as Asian, and one-fifth (19%) as Black or African American. Twelve percent of the sample identified as Hispanic or Latino, and 79% of participants reported living in a household with two or more people. Educational attainment was generally high, with 32% reporting holding a college degree and 31% reporting having attained a post-college degree (Table 1).

### 3.2. Equivalence of GDQS Tabulated from WFR Versus the GDQS App Using Cubes or Playdough

The TOST demonstrated that the GDQS computed with WFR data and the GDQS derived from the app using either the cubes or playdough method were equivalent within the pre-specified bound of 2.5 points (*p* = 0.006 for cubes and *p* < 0.001 for playdough) (Table 2). Compared to the mean GDQS calculated with WFR data (20.5 points), the mean GDQS was 2.0 and 1.3 points higher using the cubes and playdough, respectively. This difference was primarily driven by observed higher GDQS positive scores with data collected with the cubes (+2.4 points) and playdough (+1.4 points) compared to data collected with WFR (9.7 points). Small differences between methods were observed for GDQS negative. Cubes scored 0.3 points lower and playdough 0.2 points higher relative to the mean GDQS negative of 10.6 points for WFR.

### 3.3. Agreement on Classification of Risk of Poor Diet Quality Outcomes from WFR Versus the GDQS App Using Cubes or Playdough

The proportion of participants at high risk of poor diet quality outcomes was 15.9%, 10.0%, and 9.4% for dietary data collected using a WFR, GDQS app using cubes, and GDQS app using playdough, respectively. When classifying participants into low, moderate, and high-risk groups for poor diet quality outcomes, both the cubes (κ = 0.5685, *p* < 0.0001) and the playdough (κ = 0.5843, *p* < 0.0001) methods showed moderate agreement with the WFR (Table 3). Classification agreement with the WFR was 73.5% for the cubes and 75.3% for playdough.

### 3.4. Kappa Coefficients for GDQS Positive Food Groups

**Figure 1:**
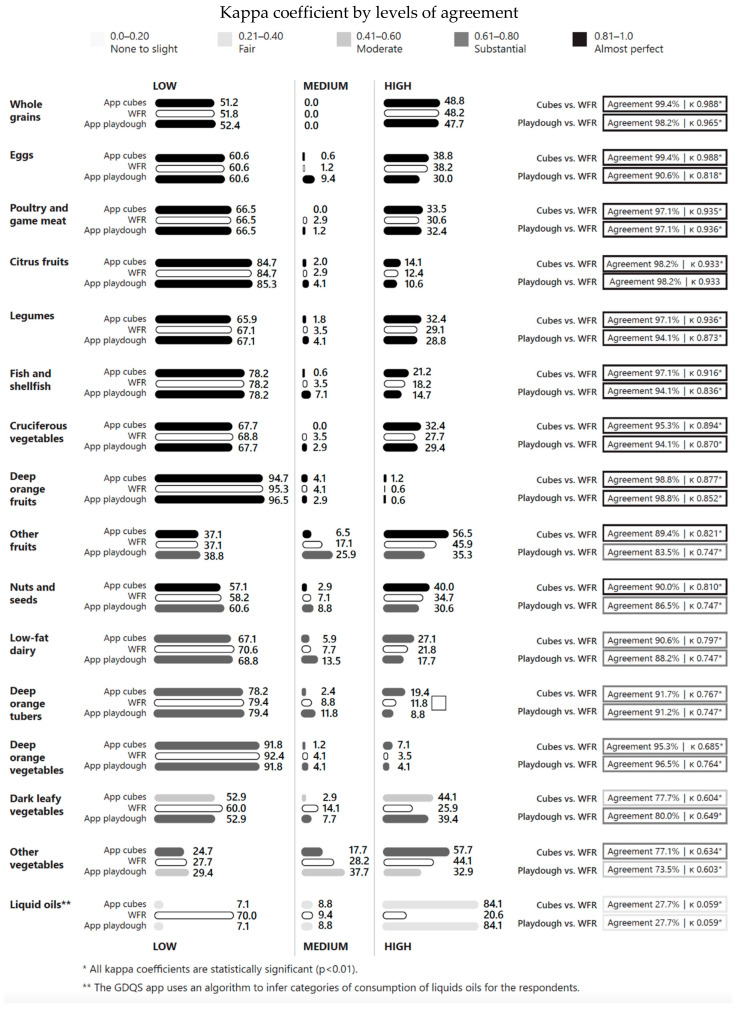
Percentage of participants consuming low, medium, or high amounts of GDQS positive food groups and levels of agreement, by data collection method.

For GDQS positive food groups, there was almost perfect agreement between the WFR and the cubes across consumption levels for nine of 16 food groups (Figure 1), substantial agreement for five food groups, and moderate for one food group (dark green leafy vegetables, κ = 0.604, 77.7% agreement) (Figure 1).

The results for playdough showed almost perfect agreement with WFR for eight of sixteen GDQS positive food groups, with substantial agreement for six groups, and moderate agreement for one food group (other vegetables, κ = 0.603, 73.5% agreement).

The liquid oils food group scored the lowest kappa coefficient of all food groups (κ = 0.059, 27.7% agreement). With the WFR, 70.0% of individuals were classified as having low consumption of liquid oils and 20.6% as high; the corresponding numbers with the GDQS app using the built-in algorithm were 7.1% and 84.1%, for low and high consumption of liquid oils, respectively.

All kappa coefficients for the GDQS positive food groups were statistically significant (*p* < 0.01).

### 3.5. Kappa Coefficients for GDQS Negative Food Groups

**Figure 2:**
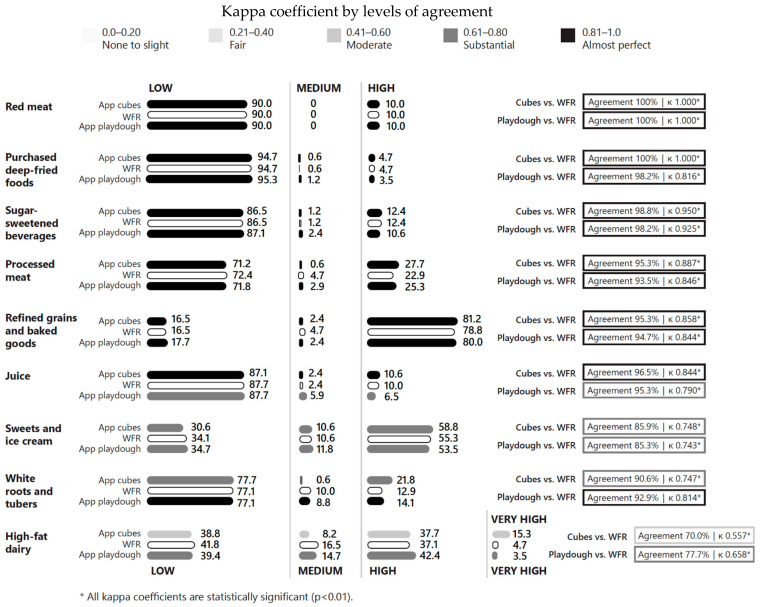
Percentage of participants consuming low, medium, high, or very high amounts of GDQS negative food groups and levels of agreement, by data collection method.

Both the cubes and playdough methods demonstrated almost perfect agreement with WFR for five out of nine GDQS negative food groups (red meat, purchased deep-fried foods, sugar-sweetened beverages, processed meat, and refined grains) (Figure 2). Substantial agreement of the two methods with WFR was observed for three GDQS negative food groups (juices, sweets and ice cream, and white roots and tubers) while high-fat diary showed substantial agreement when using playdough (κ = 0.658, 77.7% agreement) but only moderate agreement when using cubes (κ = 0.557, 70.0% agreement).

## 4. Discussion

This study showed that the two portion size estimation methods (cubes and playdough) when used with the GDQS app produced equivalent GDQS scores within the 2.5 GDQS points pre-specified level of agreement compared to data collection by the WFR. Kappa coefficients showed substantial to almost-perfect agreement for the two methods in comparison to the WFR for almost all GDQS food groups. This alignment suggests a general robustness of the app in capturing consumption category levels regardless of whether the cubes or playdough method is used.

The liquid oils food group, which was estimated using a built-in algorithm in the GDQS app, showed much lower agreement (28%) compared to other food groups. The exception for liquid oils can be attributed to two factors. The first is that the amount of liquid oils consumed may have been captured imprecisely, even with the WFR, as evidenced by the finding from the WFR data that 70% of participants consumed less than 2 g of liquid oils in the 24 h reference period. The dietary guidelines for Americans, 2020–2025, suggest that for a 2000-calorie diet, an individual should consume about 27 g of oils per day and acknowledges that most Americans already consume oil well above this level, making daily intake of less than 2 g of liquid oils extremely unlikely [29]. The second is related to the way the GDQS app categorizes liquid oils consumption. In contrast to other GDQS food groups, with the GDQS app, the quantity of consumption of liquid oils is inferred by a built-in algorithm rather than directly estimated by the respondent [15]. The algorithm used in the GDQS app to classify liquid oils consumption as low, medium, or high assumes that any mixed dish reported does contain at least some amount of liquid oils. However, upon review of the study data, many foods recorded in the GDQS app as mixed dishes did not actually contain any liquid oils.

We likely do not know the actual quantity of liquid oils consumed by study participants from either the WFR or GDQS app data. We expect that the WFR data reflect an underestimation of actual liquid oil consumption, and that the data collected with the GDQS app may reflect an overestimation of liquid oils consumption. The algorithm for classifying liquid oils consumption as low, medium, or high is one aspect of the GDQS app that warrants refinement in the future. Nevertheless, the results from this study demonstrate statistical equivalence between the WFR and the GDQS app (using cubes or playdough) for the main outcome of interest: the GDQS metric.

Since both cubes and playdough were found to be equivalent to the WFR to estimate portions at the food group level within the pre-specified equivalence margin, playdough can be considered as an alternative portion size method to collect GDQS data in addition to the cubes that are currently built into the GDQS app. However, GDQS app users should consider additional factors when selecting a method to estimate portions. It is important to note that it took twice as long on average for participants to estimate consumption levels using playdough (almost 9 min) compared to cubes (about 4 min). Other factors to consider include the cost and feasibility of procuring high-quality playdough and reliable electronic scales, as well as the need to monitor the precision of the scales and keep playdough density consistent throughout the study. It is also important to note that plastic cubes are easy to procure in most contexts (they can be 3D-printed), and are lightweight and stackable, making them more portable and durable than playdough and scales.

To our knowledge, there are no other studies that have compared the use of cubes and playdough against the WFR to estimate portion sizes at the food group level. While the concept of using playdough and other visual aids is not a novel one within the context of dietary assessment [16,19,30,31], portion size estimation at the food group level is one of the main innovations of the GDQS app. The use of cubes with the GDQS app was formally evaluated in a study conducted in Thailand [32]. In that study, data collected with the GDQS app were compared to data collected with a quantitative 24 h dietary recall instrument and data collected with the FFQ instrument. The study showed that the data collected with the GDQS app with cubes performed better than either the quantitative 24 h dietary recall instrument or the FFQ instrument in terms of the association of the GDQS with diet quality outcomes. With strong relative validity demonstrated for the GDQS app as compared to 24HR and FFQ, perhaps it is not surprising to find that the GDQS app performs equally well relative to the WFR for estimating the consumption category for each GDQS food group, and for estimating the GDQS metric, despite the methodological differences in dietary assessment methods.

Our study had strengths and limitations. One of the study’s strengths lies in its comparison against the WFR, which does not share the same limitations as the recall method employed in the GDQS app. Additionally, the study demonstrates the usefulness of an alternative portion size estimation method, the playdough, which offers the GDQS app users greater flexibility. However, this study also has its methodological limitations. Because of the COVID-19 pandemic, our study was conducted with a convenience sample, which predominantly consisted of female individuals, with two-thirds of individuals between 18 and 35 years of age, and with all individuals reporting having completed high school and living in a high-income country. Nevertheless, the inferences drawn from this sample could be extended to broader, more diverse populations in terms of age and gender, populations who may have completed less formal education, and populations living in low- and middle-income countries. We do not expect our findings to be context-limited, given that neither the use of cubes nor playdough requires context-, gender- or age-specific knowledge or complex numerical skills.

Evidence on the feasibility of respondents using cubes to assess the level of consumption for GDQS food groups is available from a formative study carried out in Ethiopia [33]. In that study, 78% of respondents administered the GDQS app with cubes reported that they did not find it difficult to assess amounts consumed at the food group level using the cubes as part of a follow-up questionnaire to obtain respondent feedback about the GDQS app interview. Two-thirds of respondents in that study reported that they did not complete primary school.

## 5. Conclusions

In conclusion, this study provides compelling evidence for the equivalence of the GDQS app using either the cubes or playdough method with the WFR in assessing diet quality as measured by the GDQS metric. The substantial or almost perfect agreement observed across 22 of the 25 GDQS food groups for cubes and 23 of the 25 for playdough underscores the potential of these innovative tools in simplifying dietary assessment while maintaining accuracy. The necessary improvement of the liquid oils algorithm in the GDQS app demonstrates the iterative nature of refining such methodologies. The numerous examples of successful cross-cultural investigations suggest applicability across diverse populations. These findings contribute to the growing body of research validating simplified tools and metrics and pave the way for more frequent and accessible evaluations of diet quality to better inform policies and programs globally.

## Figures and Tables

**Table 1:** Sample characteristics.

Characteristics of Study Participants	*n*	Percentage
Age groups		
18–24 y	48	28
25–35 y	69	41
36–45 y	21	12
46–73 y	32	19
Sex (%)		
Male	48	28
Female	121	71
Refused to answer	1	1
Race of participant		
White	86	51
Black or African American	33	19
Asian	40	24
American Indian or Alaska Native	1	1
Other	10	6
Hispanic or Latino		
Yes	20	12
No	150	88
Number of people living in the household		
1	36	21
2	43	25
3	33	19
4 or more	58	34
Highest level of education completed		
High school completed	0	0
Some college	26	15
College degree	55	32
Some post-college coursework	36	21
Post-college degree	53	31

**Table 2:** Equivalence of GDQS computed using data from the weighed food records and the GDQS app, by portion size estimation method.

	WFR	GDQS App with Cubes	GDQS App with Playdough	Paired Difference Cubes-WFR			Paired Difference Playdough-WFR			Equivalence WFR vs. Cubes	Equivalence WFR vs. Playdough
	Mean (SE)			Mean (95% CI)	Min.	Max.	Mean (95% CI)	Min.	Max.		
GDQS	20.5 (0.4)	22.5 (0.4)	21.8 (0.4)	2.0 (1.7, 2.4)	−4.0	9.3	1.3 (1.0, 1.7)	−4.8	8.3	Yes (overall *p* = 0.006)	Yes (overall *p* < 0.001)
GDQS positive	9.7 (0.4)	12.2 (0.4)	11.1 (0.4)	n/a	n/a	n/a	n/a	n/a	n/a	n/a	n/a
GDQS negative	10.6 (0.2)	10.3 (0.2)	10.8 (0.2)	n/a	n/a	n/a	n/a	n/a	n/a	n/a	n/a

WFR, weighed food records; SE, standard error; CI, confidence interval; GDQS, Global Diet Quality Score, n/a, non applicable.

**Table 3:** Kappa coefficients of agreement for risk of poor diet quality outcomes, by portion size estimation method.

	WFR	GDQS App with Cubes	GDQS App with Playdough
Risk of poor diet quality outcomes (N, %)			
Low (GDQS ≥ 23)	54 (31.8%)	83 (48.8%)	69 (40.6%)
Moderate (GDQS ≥ 15 and <23)	89 (52.4%)	70 (41.2%)	85 (50.0%)
High (GDQS < 15)	27 (15.9%)	17 (10.0%)	16 (9.4%)
Kappa coefficient of agreement with WFR	n/a	κ = 0.5685, *p* < 0.001 (moderate agreement)	κ = 0.5843, *p* < 0.001 (moderate agreement)
Agreement with WFR (%)	n/a	73.5%	75.3%

WFR, weighed food records; SE, standard error; CI, confidence interval; GDQS, Global Diet Quality Score, n/a, non applicable.

## Data Availability

The raw data supporting the conclusions of this article will be made available by the authors on request.

## Abbreviations

The following abbreviations are used in this manuscript:

MDPI	Multidisciplinary Digital Publishing Institute
DOAJ	Directory of open access journals
FHI	Family Health International
GDQS	Global Diet Quality Score
NCD	Non-communicable disease
TOST	Two one-sided t-test
WFR	Weighed food records
INSP	Institute of Public Health
